# The Effect of Metformin in Diabetic and Non-Diabetic Rats with Experimentally-Induced Chronic Kidney Disease

**DOI:** 10.3390/biom11060814

**Published:** 2021-05-30

**Authors:** Mohammed Al Za’abi, Badreldin H. Ali, Yousuf Al Suleimani, Sirin A. Adham, Haytham Ali, Priyadarsini Manoj, Mohammed Ashique, Abderrahim Nemmar

**Affiliations:** 1 Department of Pharmacology and Clinical Pharmacy, College of Medicine and Health Sciences, Sultan Qaboos University, Khoud 123, Oman; zaabi@squ.edu.om (M.A.Z.); alibadreldin@hotmail.com (B.H.A.); yousufm@squ.edu.om (Y.A.S.); priyadarsinimanoj@gmail.com (P.M.); mohammed.ashique@squ.edu.om (M.A.); 2 Department of Biology, College of Science, Sultan Qaboos University, Muscat 123, Oman; sadham@squ.edu.om; 3 Department of Animal and Veterinary Sciences, College of Agricultural and Marine Sciences, Sultan Qaboos University, Muscat 123, Oman; h.ali@squ.edu.om; 4 Department of Physiology, College of Medicine and Health Sciences, United Arab Emirates University, Al Ain, United Arab Emirates

**Keywords:** adenine, chronic kidney disease, diabetes, metformin, rats

## Abstract

This work aimed to investigate whether treatment with the antidiabetic drug metformin would affect adenine-induced chronic kidney disease (CKD) in non-diabetic rats and rats with streptozotocin (STZ)-induced diabetes. Rats were randomly divided into eight groups, and given either normal feed, or feed mixed with adenine (0.25% *w/w*, for five weeks) to induce CKD. Some of these groups were also simultaneously treated orally with metformin (200 mg/kg/day). Rats given adenine showed the typical signs of CKD that included detrimental changes in several physiological and traditional and novel biochemical biomarkers in plasma urine and kidney homogenates such as albumin/creatinine ratio, N-acetyl-beta-D-glucosaminidase, neutrophil gelatinase-associated lipocalin, 8-isoprostane, adiponectin, cystatin C, as well as plasma urea, creatinine, uric acid, indoxyl sulfate, calcium, and phosphorus. Several indices of inflammation and oxidative stress, and renal nuclear factor-κB and nuclear factor erythroid 2-related factor 2 levels were also measured. Histopathologically, adenine caused renal tubular necrosis and fibrosis. The activation of the intracellular mitogen-activated protein kinase signaling pathway was inhibited in the groups that received metformin and STZ together, with or without adenine induced-CKD. Induction of diabetes worsened most of the actions induced by adenine. Metformin significantly ameliorated the renal actions induced by adenine and STZ when these were given singly, and more so when given together. The results suggest that metformin can be a useful drug in attenuating the progression of CKD in both diabetic and non-diabetic rats.

## 1. Introduction

Chronic kidney disease (CKD) refers to a group of chronic progressive diseases that are characterized by increasing prevalence rates (currently about 14%) and high mortality that poses serious global threats to human life and health [1]. CKD can progress to end stage renal disease (ESRD) that requires expensive renal transplantation or dialysis. There are currently no specific treatments that target the etiologies of CKD, and therefore there is still a pressing need to search for new agents for this disease, including novel and old repurposed drugs [2,3,4].

The adenine-induced CKD model in rats and mice is a commonly used method for creating a metabolic abnormality that closely mimics the disease in humans. In this model, adenine is given to these animals in the feed at different concentrations for variable periods [5,6]. The excretion of nitrogenous compounds in the adenine-treated animals is diminished by renal tubular occlusion due to the formation of 2,8-dihydroxyadenine crystals, leading to accumulation of various guanidino compounds (such as methylguanidine and guanidinosuric acid) and urea nitrogen in blood [7]. Adenine-induced CKD causes inflammation, oxidative, and nitrosative stress, and alters the composition of the microbiota [8,9,10]. Several drugs and dietary supplements have been tested in this model for their efficacy against experimental CKD [11,12].

We have previously reported that streptozotocin (STZ)-induced diabetes worsens most of renal function tests in rats. Administration of adenine (to induce CKD) in STZ-diabetic rats aggravates further the renal damage induced by adenine alone [13]. Diabetes is also known to worsen CKD in humans [14].

The biguanide metformin is currently the first-line oral drug for treating type-2 diabetes mellitus [15]. It produces its antidiabetic action through several mechanisms [16,17]. Metformin has also been reported to prevent fibrosis in several organs, including the kidneys [18,19]. In addition to treatment of diabetes, the drug has also been reported to be beneficial in treating several other diseases and conditions that include lung and breast cancers [20,21], inflammatory skin disorders [22], and neurological diseases [23]. 

In the present work, we have tested the possible ameliorative effects of metformin on adenine-induced CKD in diabetic and non-diabetic rats. While this work was being prepared for publication, a paper on the effect of metformin on the surgical (subtotal nephrectomy) model of CKD has been reported in non-diabetic rats [24]. However, as far as we are aware, the effect of metformin has not been tested before in the adenine model of CKD in diabetic and non-diabetic rats.

## 2. Materials and Methods

### 2.1. Animals

Wistar rats (190–200 g) were obtained from the Small Animal House of the Sultan Qaboos University and were housed in a room with controlled environment (a temperature of 22 ± 2 °C, relative humidity of about 60%, with a 12 h light–dark cycle), and were provided ad libitium with additive-free standard diet (Oman Flour Mills, Muscat, Oman), and tap water. 

### 2.2. Induction of Diabetes

Rats were rendered diabetic by an intraperitoneal (i.p.) injection of STZ (55 mg/kg) dissolved in 0.1 M citrate buffer (pH 4.5). Other animal groups were injected with citrate buffer. Seventy-two hours after STZ injection, a drop of blood was taken from the tail vein and fasting blood glucose level was measured using a blood glucose monitoring system (One Touch^®^ UltraMini^®^, Life Scan, Inc., Milpitas, CA, USA). Rats with blood glucose level >20 mmol/L were considered diabetic. Treatments (for 35 days) were started three weeks after STZ injection.

### 2.3. Experimental Design

The animals (*n* = 48) were randomly distributed into eight equal groups and treated as follows for 35 consecutive days:

1. Control (CON) group continued to receive the same diet without treatment until the end of the study.
2. Adenine (A) group was switched to a powdered diet containing adenine (0.25% *w/w* in feed given daily).
3. Diabetes (STZ) group was induced by injecting the rats i.p. with STZ, as described above.
4. Adenine + Diabetes (A + STZ) group was treated with adenine and STZ, as mentioned in the second and third groups.
5. Metformin (MF) group was treated daily by oral gavage with metformin (200 mg/kg/day) dissolved in distilled water.
6. Adenine + Metformin (A + MF) group was treated with adenine and metformin as mentioned in the second and fifth groups.
7. Diabetes + Metformin (STZ + MF) group was treated with STZ and metformin, as mentioned in the third and fifth groups.
8. Adenine + Diabetes + Metformin (A + STZ + MF) group was treated with adenine, STZ and metformin, as mentioned in second, third and fifth groups, respectively.

One day before the rats were sacrificed, urine of each rat was collected over a 24-h period, and its volume measured. Immediately after the end of the treatment period, rats were anesthetized with a combination of ketamine (75 mg/kg) and xylazine (5 mg/kg) given i.p. injection. Blood was then collected from the inferior vena cava in heparinized tubes and centrifuged at 900× *g* for 15 min, at 4 °C to separate plasma. The plasma harvested was stored frozen at −80 °C pending biochemical analyses within 10 to 21 days. The rats were then sacrificed by an overdose of anesthesia. The kidneys were removed from the rats, washed with ice-cold saline, blotted with a piece of filter paper, and weighed. A small piece from the right kidney was fixed in 10% buffered formalin pending histological analysis. The remainder of the right and left kidneys were individually wrapped in aluminum foil and then dipped in liquid nitrogen and stored at −80 °C, pending analysis within about one to three weeks.

### 2.4. Drugs, Chemicals, and Biochemical Analysis

Adenine and STZ were obtained from Sigma (St. Louis, MO, USA). Metformin was a gift from the National Pharmaceutical Industries (Muscat, Oman). The rest of the chemicals were of the highest purity grade available. Urea, uric acid, calcium, phosphorus, and albumin were measured using an automated biochemical analyzer, Mindray BS-120 chemistry analyzer, from Shenzhen Mindray Bio-Medical Electronics Co. (Shenzhen, China). Creatinine, Superoxide dismutase (SOD), glutathione reductase (GR), total antioxidant capacity (TAC), and *N*-acetyl-β-D-glucosaminidase (NAG) were measured by colorimetric method, using BioVision kit (Milpitas, CA, USA). Indoxyl sulfate, 8- isoprostane and nuclear factor erythroid 2-related factor 2 (Nrf2) were measured using ELISA kits from MyBioSource, Inc. (San Diego, CA, USA). NF-κB (Nuclear Factor-kappa B), 8-hydroxy-2-deoxy guanosine (8-OHdG) and transforming growth factor (TGF-β1) were measured using ELISA kit from Cusabio Biotech Co. Ltd. (Wuhan, Hubei, China). The ELISA kits for measuring cystatin C, interleukin-1β (IL-1β), adiponectin, interleukin-6 (IL-6), and neutrophil gelatinase-associated lipocalin (NGAL) were obtained from Thermo Fisher Scientific, Inc. (Waltham, MA, USA). Interleukin-10 (IL-10) was measured using ELISA kits from Abcam (Cambridge, UK). Osmolality was measured by the freezing point depression method using the Osmomat 3000 osmometer (Gonotec GmbH, Berlin, Germany).

### 2.5. Histopathological Analysis

Formalin-fixed renal tissues were dehydrated, cleared in xylene and paraffin, and embedded using standard technique. Sections were cut by a rotary microtome at 4 μm thickness and were stained by hematoxylin and eosin (to evaluate acute tubular necrosis), Periodic Acid–Schiff (to assess glomerular integrity) and Picro-Sirius red (to assess interstitial fibrosis). Details of the techniques used were reported before (Ali et al., 2018). Sections were examined blindly under a light microscope by a histopathologist unaware of the treatments given. The percentage of renal tubular necrosis was scored by semi-quantitative method, as previously described by Ali et al., [5], on a scale 0–4 as following; 0 = normal, no necrosis; 1 < 10%; 2 = 10–25%; 3 = 26–75%; 4 > 75%. Three 40X fields were evaluated from each kidney section of each animal of the 8 groups and the mean percentage was converted to the score value. Sirius red stained slides were analyzed following the procedure described by Manni et al. [25]. The slides were examined by Olympus B51X microscope attached to Olympus DP70 camera, and images were acquired using the x40 objective lens. Three random images of the renal cortex were acquired from each kidney of each animal of the eight groups and stored as TIFF 24 bit RGB color image files. Image analysis was performed on the stored images using ImageJ^®^ image analysis software. Briefly, the images were converted into grey scale and the red stained collagen was isolated using the hue histogram filter available in “Threshold Color” followed by measuring the isolated area as a percentage. Fibrosis index (%) assessed the collagen content of the tissues [26,27], by calculating the ratio of the mean Sirius red-stained positive area to the mean whole area of each section for each animal.

### 2.6. Western Blotting

The activation of the signaling pathway of intracellular mitogen-activated protein kinase (MAPK) was assessed by western blotting as described before [28].

### 2.7. Statistical Analysis

Data were given as mean ± SEM and were analyzed by one-way analysis of variance followed by Bonferroni’s multiple comparison test (GraphPad Prism version 5.03, San Diego, CA, USA); *p* < 0.05 was considered statistically significant.

## 3. Results

### 3.1. Physiological Data

Table 1 shows some physiological data of all the groups of rats in the experiment. metformin, adenine, and STZ, given either separately or together, significantly decreased the weight gain of rats, and significantly increased the absolute and relative kidney weight, feed, and water intake and urine output.

**Figure 1:**
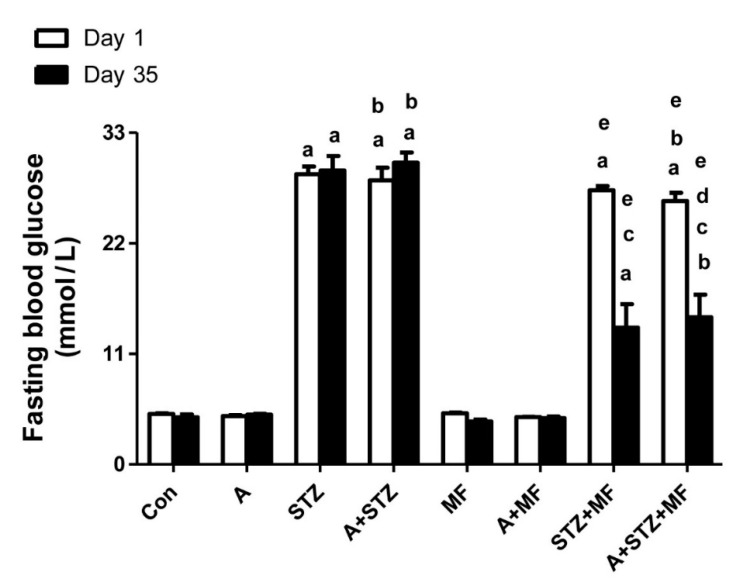
The fasting blood glucose level in control rats, and rats treated with metformin (MF), adenine (A) and streptozotocin (STZ) alone or in combination. Each column and vertical bar represent mean ± SEM (*n* = 6). Differences between the groups were assessed by one-way analysis of variance (ANOVA) followed by Bonferroni’s multiple comparison test, where *p* < 0.05. ^a^ denotes significant difference between control group vs. different groups; ^b^ denotes significant difference between the group given A alone vs. other groups treated with A; ^c^ denotes significant difference between the group given STZ alone vs. other groups treated with STZ; ^d^ denotes significant difference between the group given A + STZ vs. other groups treated with A + STZ; ^e^ denotes significant difference between the group given MF only vs. other groups treated with MF.

Figure 1 depicts the concentrations of fasting blood glucose (FBG) in rats following treatments with either adenine, STZ, or metformin, each given separately or in combination. STZ significantly increased the FBG, and treatment with adenine alone did not cause a significant affect. Rats that received STZ, with or without adenine had significantly higher FBG concentration when compared with the control rats, and with rats treated with adenine alone. The FBG level was significantly decreased in diabetic rats treated with metformin alone, and with metformin plus adenine.

### 3.2. Renal Variables

Table 2 shows the effects of treatments with adenine, STZ, and metformin, given singly or together on some renal function tests in plasma. Compared with controls, rats fed adenine had significantly higher urea, creatinine, uric acid, and phosphorus, and lower calcium concentrations. STZ given alone or with adenine had similar biochemical changes to that of adenine, except that both phosphorus and calcium were significantly increased in diabetic rats. Results in the group receiving metformin alone did not significantly differ from that of the control. Treatment with metformin significantly reduced the effects of adenine and STZ (either given singly or together). The effect of treatment with adenine, STZ, or their combinations, on some urinary parameters are shown in Table 3. Adenine and STZ treatments significantly increased albumin/creatinine levels, as well NAG activity, and significantly decreased osmolality and creatinine clearance. In normal rats, metformin treatment was without significant action on any of these indices. When metformin was given together with either adenine or STZ, or both of them, the changes in the measured analytes were significantly abated.

Effect of metformin treatment on some plasma cytokines (IL-1β, IL-6, IL-10, and TGF -β1) in STZ diabetic and non-diabetic rats with adenine-induced CKD are shown in Table 4. Metformin alone had no significant effect on the parameters measured. Treatments of rats with adenine or STZ significantly increased the concentrations of these parameters when compared with the controls or the metformin-treated rats. This action was augmented further when adenine and STZ were given together. Treatment with metformin alone significantly reduced the actions of adenine or STZ on the measured cytokines. Treatment with metformin in rats that have been given both adenine and STZ significantly reduced the concentrations of the four parameters measured, when compared with values obtained from the rats treated either with adenine or STZ, alone or in combination.

As shown in Table 5, treatment with both adenine and STZ (each given singly) significantly decreased the renal oxidative stress biomarkers (GR, SOD, and CAT), and increased that of 8-isoprostane and 8-OHdG in plasma, when compared with their values in the control or the metformin-treated group. Combination of these two agents significantly aggravated this action. Treatment with metformin in combination with either adenine or STZ, or both of them, has significantly alleviated these effects.

**Figure 2:**
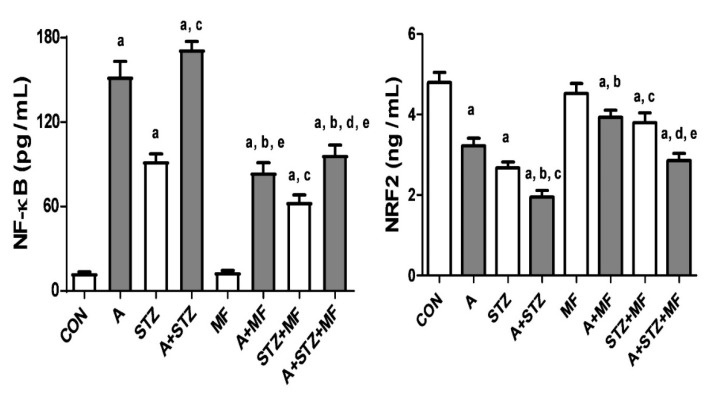
The renal concentration of nuclear factor-kappa B (NF-κB) and nuclear factor erythroid 2-related factor 2 (Nrf2) in control rats, and rats treated with metformin (MF), adenine (A), and streptozotocin (STZ) alone or in combination. Each column and vertical bar represent mean ± SEM (*n* = 6). Differences between the groups were assessed by one-way analysis of variance (ANOVA) followed by Bonferroni’s multiple comparison test, ^a^ denotes significant difference between control group vs. different groups; ^b^ denotes significant difference between the group given A alone vs. other groups treated with A; ^c^ denotes significant difference between the group given streptozotocin (STZ) alone vs. other groups treated with STZ; ^d^ denotes significant difference between the group given A + STZ vs. other groups treated with A + STZ; ^e^ denotes significant difference between the group given MF only vs. other groups treated with MF.

Figure 2 depicts the effects of treatment with adenine, STZ, and metformin, each given singly or in combination, on the levels of NF-*κ*B or Nrf2 in the renal homogenates. Nrf2, was found to be significantly decreased in adenine-treated rats, and in rats with STZ-induced diabetes. Concomitant treatment with metformin in these latter groups was significantly effective in ameliorating the decrease in Nrf2 level. The opposite actions were noted with NF-*κ*B level in all the experimental groups.

### 3.3. Histopathology Results

**Figure 3:**
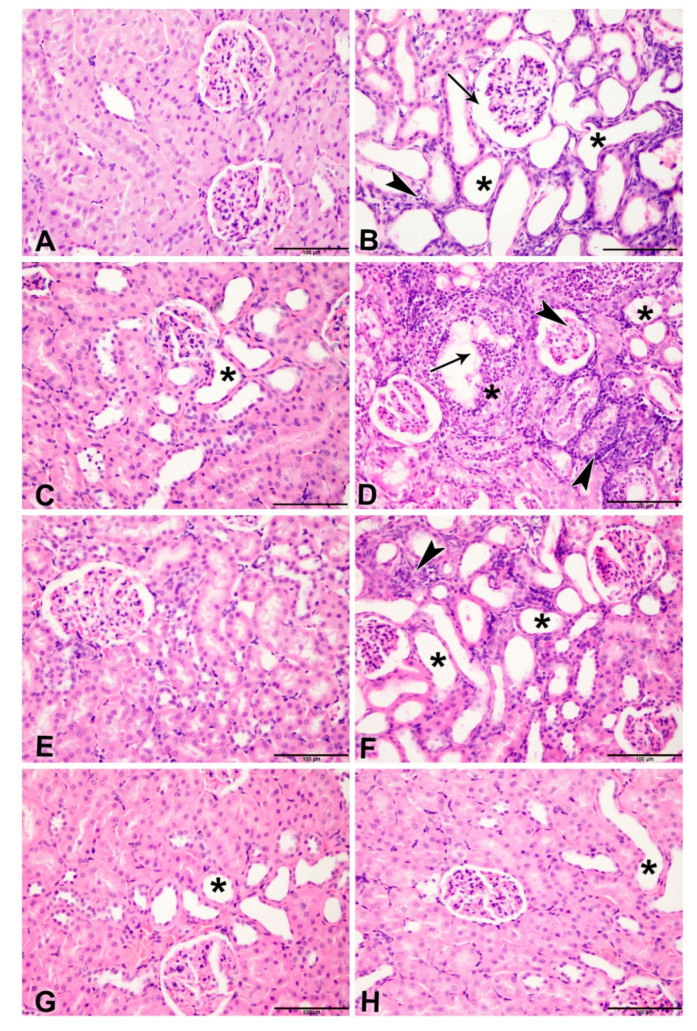
Representative light microscopy images of H&E-stained kidneys sections (Bar = 100 µm). (**A**) Control group showing normal structures and architecture of renal tissue with intact glomeruli and renal tubules (Score 0); (**B**) rats treated with adenine (A) showing cystic dilatation of multiple renal tubules (asterisks), renal tubular necrosis with pyknotic nuclei (arrowheads), marked basophilia, and dilatation of bowman’s capsule (arrow) (Score 4); (**C**) rats treated with streptozotocin (STZ)-induced diabetes showing group cystic dilation of few renal tubules (asterisk) (Score 2); (**D**) rats treated with adenine and STZ showing, renal tubular necrosis (arrowheads), cystic dilatation of renal tubules (asterisks), and cellular casts in renal tubules (arrow) (Score 4); (**E**) rats treated with metformin (MF) showing normal glomerulus and renal tubules (Score 0); (**F**) rats treated with adenine and MF showing renal tubular dilatations (asterisks) and tubular necrosis with marked basophilia (arrowhead) (Score 3); (**G**) rats treated with STZ and MF showing normal histological structures of the majority of the renal tubules, intact glomeruli and few dilated tubules (asterisk) (Score 1); (**H**) rats treated with adenine, STZ and MF showing normal renal tubules and intact glomeruli with few dilated tubules (Score 0).

**Figure 4:**
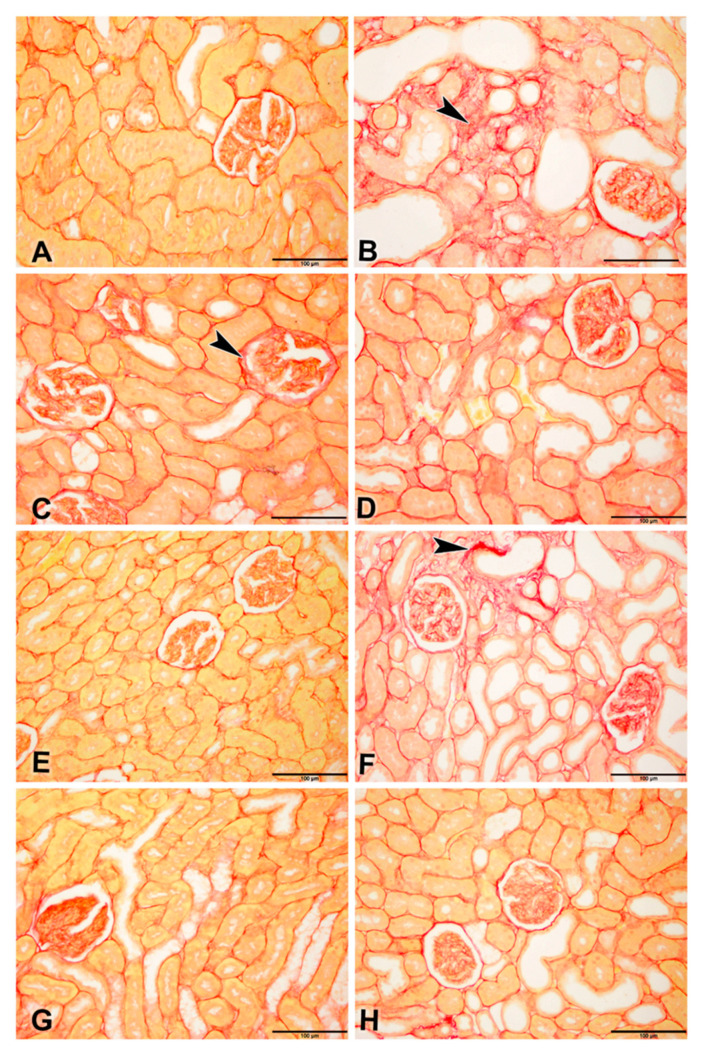
Representative photographs of Picro-Sirius red staining on sections of renal tissues (Bar = 100 µm) from control rats and rats treated with adenine (A), streptozotocin (STZ), and metformin (MF), alone or in combination. (**A**) Control and (**E**) MF groups showed normal kidney architecture and histology. (**B**) adenine (**A**,**C**) STZ showed stained fibrotic areas fibers (arrowheads) and yellow non-collagen structures. (**D**) A + STZ groups showed changes in normal kidney architecture and histology. (**F**) The A + MF group showed fibrotic areas fibers (arrowheads). (**G**) STZ + MF and (**H**) A + STZ + MF groups showed an improvement in the histologic appearance and a significant decrease in fibrotic areas fibers when compared with the STZ and A + STZ group, respectively.

**Figure 5:**
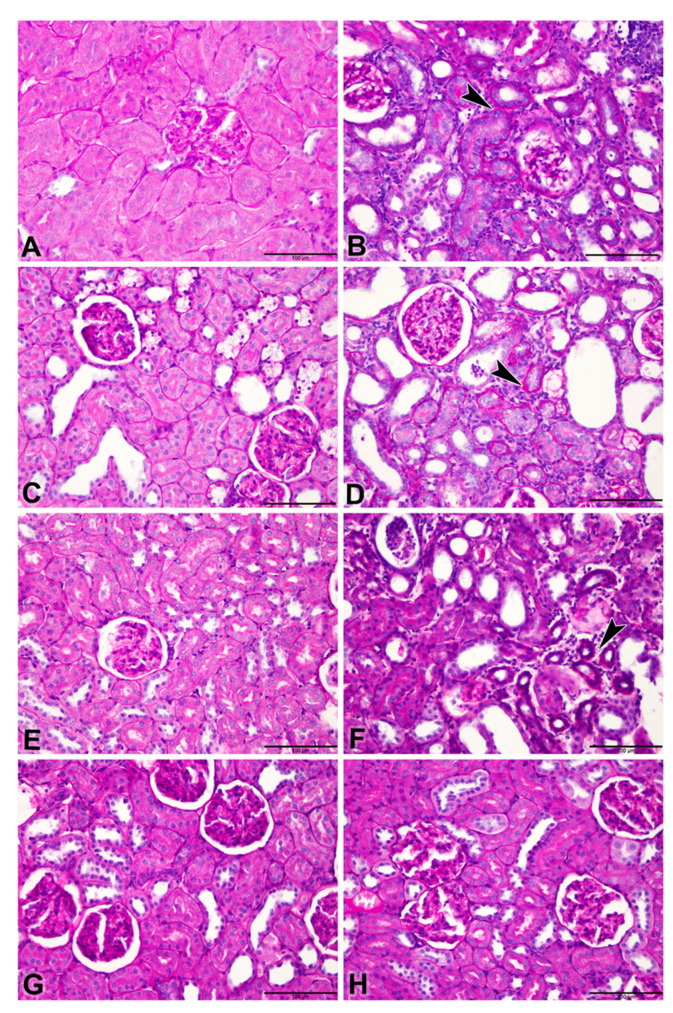
Representative photographs of Periodic Acid–Schiff (PAS) staining on sections of renal tissues (Bar = 100 µm) from control rats and rats treated with adenine (**A**), streptozotocin (STZ), and metformin (MF), alone or in combination. Control rats and rats treated with MF only ((**A**,**E**), respectively) showed normal renal histology. (**B**) Adenine (**A**,**D**) A + STZ alone treated groups shows tubular atrophy represented by a thickened tubular basement membrane (arrow) stained positive (magenta red). (**C**) STZ (STZ) group shows only a slight change in tubular atrophy, when compared to control group. (**F**) Adenine-MF (A + MF) group showing a thickened tubular basement membrane (arrow). (**G**) STZ + MF and (**H**) A + STZ + MF groups showed improvement in the histologic appearance and a significant decrease in tubular atrophy.

Table 6 and Figure 3, Figure 4 and Figure 5 show the effect of metformin (MF) administration on histopathology of kidney section of diabetic and non-diabetic rats with adenine-induced CKD. Examined renal tissue sections from rats in group 1 (control) showed normal structures and architecture of renal tissue with intact glomeruli and renal tubules (0.00 ± 0.0%, Score 0) (Figure 3A). Sections in group 2 (adenine) showed cystic dilatation of multiple renal tubules, renal tubular necrosis in 81.1 ± 2.22% of the examined tissue areas (Score 4) with pyknotic nuclei, marked basophilia, and dilatation of Bowman’s capsule (Figure 3B). Examined renal tissues from rats in group 3 (STZ) exhibited cystic dilation of few renal tubules (20.6 ± 2.0%, Score 2) (Figure 3C). Examined renal tissue sections from rats in group 4 (adenine + STZ) revealed renal tubular necrosis, cystic dilatation of renal tubules, and cellular casts in renal tubules (78.9 ± 3.30%, Score 4) (Figure 3D). Tissue sections of group 5 (metformin) showed normal glomerulus and renal tubules (0.6 ± 0.56%, Score 0) (Figure 3E). In group 6 (adenine + metformin) the kidney sections revealed renal tubular dilatations and tubular necrosis with marked basophilia (48.9 ± 1.41%, Score 3) (Figure 3F). Examined sections of rats in group 7 (STZ + MF) exhibited normal histological structures of the majority of the renal tubules, intact glomeruli, and few dilated tubules (7.8 ±1.11%, Score 1) (Figure 3G). Renal tissues of animals in group 8 (adenine + STZ + metformin) showed normal renal tubules and intact glomeruli with few dilated tubules (0.00 ± 0.0%, Score 0) (Figure 3H).

The scores of the renal tubular necrosis and the fibrosis index percentage are summarized in Table 6.

Picro-Sirius red-stained renal tissues from different groups showed the distribution of Sirius red-stained fibrotic areas fibers and yellow non-collagen structures (Figure 4A–H).

Periodic Acid–Schiff (PAS) stained renal tissues showed tubular atrophy (Figure 5A–H).

### 3.4. Western Blotting

**Figure 6:**
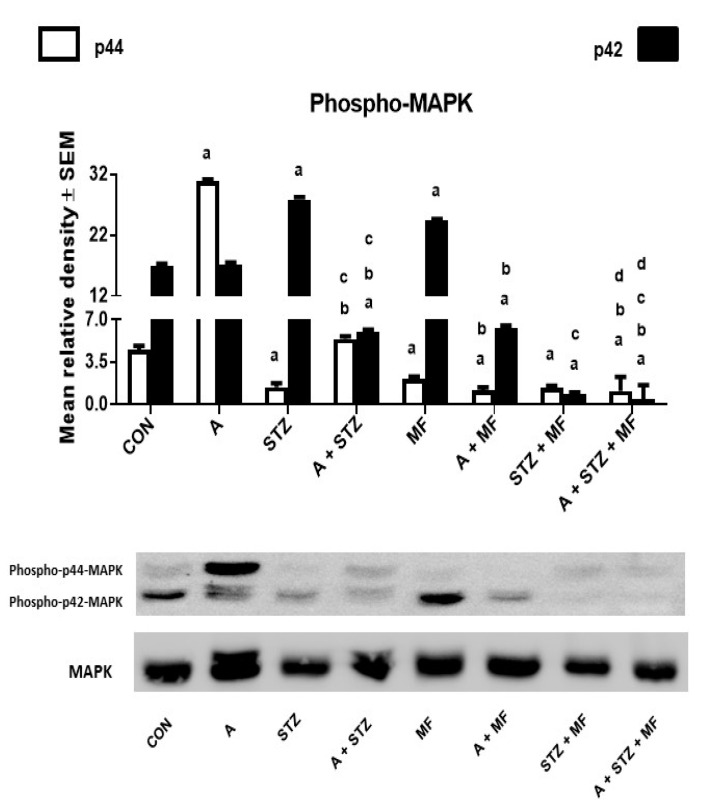
The renal concentration of phospho-p44 or phospho-p42 mitogen-activated protein kinase (MAPK) in control (CON) rats, and those treated with adenine (A, 0.25% *w/w* in feed), streptozotocin (STZ, 55 mg/kg by intraperitoneal injection), metformin (MF, 200 mg/kg/day), or a combination of these treatments. Each vertical column with bar represents the mean ± SEM (from 6 rats in each group). Differences between the groups were assessed by one-way analysis of variance (ANOVA) followed by Bonferroni’s multiple comparison test, where *p* < 0.05 was considered significant. ^a^ denotes significant difference between control group vs. different groups; ^b^ denotes significant difference between the group given A alone vs. other groups treated with A; ^c^ denotes significant difference between the group given STZ alone vs. other groups treated with STZ; ^d^ denotes significant difference between the group given A + STZ vs. other groups treated with A + STZ; ^e^ denotes significant difference between the group given MF only vs. other groups treated with MF.

The results of the western blot analysis of rat kidney homogenates (Figure 6) show the intensity of the phosphorylated MAPK on both sites (p44 and p42). Adenine significantly increased the levels of the MAPK, notably on the p44 site, and this effect was significantly reduced when both metformin and STZ were given together. The phosphorylation on p42 site was apparent in control, and metformin treated rats. 

## 4. Discussion

The present results showed that the rats that have been fed a diet mixed with adenine exhibited all the previously reported physiological, biochemical, histopathological, and molecular effects that occur in adenine-induced CKD [5,10]. The results also confirmed that STZ-induced diabetes aggravated these actions in rats with adenine-induced CKD [13]. We have experimentally shown here that metformin treatment significantly mitigates the actions of CKD in diabetic and non-diabetic rats.

In the present work we have used metformin at a dose of 200 mg/kg/day, which is known to be a safe dose for rats and does not elevate lactate concentration in the blood [29,30]. Metformin at this dose did not cause any significant alterations in renal function or structure. Similar findings have been found by others before [31].

Metformin significantly elevated urinary albumin/creatinine ratio in rats with adenine-induced CKD and STZ-induced rats. The mechanism of this action was not certain, but it is possibly related to change in glomerular filtration, decreased blood glucose levels, blood pressure, and degree of insulin resistance and/or renal proximal tubular impairment [32]. NAG is a hydrolytic lysosomal enzyme, found predominantly in the proximal tubules [33] and is considered one of the most important and commonly used indices of tubular damage, mainly because NAG assays are sensitive enough to allow dilution of the urine, thus overcoming any enzyme inhibition [34,35]. NAG activity in urine was significantly increased by adenine and STZ when given singly or together. In rats given adenine and STZ (singly or together), metformin treatment was effective in significantly mitigating the increased NAG activity. Urine osmolality was markedly and significantly decreased in rats with adenine-induced CKD, and this was mitigated by metformin. It has been shown that sustained high urine volume and low urinary osmolality are independent risk factors for quicker decline in glomerular filtration rate in patients with CKD [36,37]. Metformin significantly mitigated the actions of adenine and STZ, which is in line of the report of Efe et al. [38].

Increased production of reactive oxygen species (ROS) and/or reduced antioxidant defense capacity), apoptosis, and inflammation are established to be involved in the pathogenesis of CKD in humans and experimental animals [5,39,40,41]. Imbalances in the ROS are known to be important drivers in the inflammatory process [42]. These actions may be the underlying basis of the consequent cardiovascular and other health outcomes of CKD [43,44]. Treatment with adenine in this work caused the expected and previously reported actions on the biomarkers of oxidative stress and inflammation in plasma, urine, and kidney homogenates [8,45]. Our present results indicated that metformin treatment significantly increased the plasma concentration of the indices of the oxidative stress and reduced the pro-inflammatory mediators. These actions support the reports that metformin is a strong antioxidant and anti-inflammatory agent [22,45].

Notable among the biochemical findings in the present work is that adenine feeding significantly increased the key profibrotic growth factor TGF-β1 level in the kidneys. In a previous work, we have shown that TGF-β1 is also increased in other tissues, such as gastrointestinal mucosa [9,46]. This has also been reported by others [47]. TGF-β1 signaling pathway is established to be activated in CKD and promote renal fibrosis [48]. In this work, metformin significantly antagonized the increase in TGF-β1. This property of metformin was reported before [49]. This action of metformin may explain the decreased level of renal fibrosis seen histopathologically in this work. Metformin direct renoprotective action, independent of its glucose lowering effect, has been reported before in other models of renal disease such as peri-renal adipose inflammation-induced renal dysfunction [50], and unilateral ureteral obstruction [51].

The transcription factor Nrf2, is known to regulate the expression of more than 200 cytoprotective genes encoding antioxidant proteins, induce antioxidant enzymes to respond to oxidative stress, and antagonize oxidative and inflammatory damage [42,52]. NF-*κ*B is a nuclear transcription factor that has a significant role in many pathophysiological processes that includes inflammation, oxidative stress, immune reaction, and apoptosis [53]. In this work, renal Nrf2, measured by an ELISA method, was found to be significantly decreased in adenine-treated rats, and in rats with STZ-induced diabetes. Concomitant treatment with metformin in these latter groups was significantly effective in ameliorating the decrease in Nrf2 level. The opposite was found with NF-*κ*B level in all the experimental groups. This finding is in line with the results of indices of oxidative stress and inflammation in these groups.

In the present adenine model of CKD, metformin produced a similar ameliorative action as in the subtotal nephrectomy model of CKD [24]. However, in this investigation we extended our study by including diabetic and non-diabetic rats. Further, we used here more traditional and novel biomarkers to assess the effect of metformin on adenine-induced CKD. The latter included indoxyl sulfate, which is a reliable uremic toxin, especially for early-stage CKD [54], and 8-OHdG [55].

It has recently been reported that in non-diabetic mice with partial nephrectomy-induced CKD, metformin protects against experimental acute kidney disease (AKI) but not against AKI-CKD progression [56]. It was found in a retrospective analysis that type-2 diabetic patients with CKD stage 5 who took metformin were less likely to develop ESRD than those who do not use it [57]. More recently, a systematic and meta-analysis concluded that taking metformin is associated with significantly less risks of all-cause mortality and cardiovascular events in type-2 diabetic patients with mild to moderate CKD [12].

It is well known that inflammation and apoptosis are regulated by MAPK pathway. We assessed the role of metformin on MAPK activation by determining the phosphorylation of ERK1/2 at p44 and p42 sites. Adenine-induced CKD resulted in activation of MAPK treatment, and when it was given to animals with metformin, the phosphorylation of MAPK p44 in renal tissue was significantly mitigated. Thus, there was attenuation of apoptosis and inflammation in renal tissue of animals that were treated with metformin. Therefore, we concluded that metformin alleviated kidney inflammation and apoptosis protecting against adenine induced-CKD via inhibiting NF-κB p65 and MAPK signaling pathways, and that their increased levels are associated with injured kidneys [58].

A limitation of this study is that it used one dose of metformin (based on previously-published literature). Using three or more doses would have provided useful dose response data for all the analytes measured here. However, this was not feasible due to technical and financial limitations. Notwithstanding this limitation, the study has shown, using various biochemical, histopathological, histochemical, and molecular techniques, that metformin, at the dose used, was effective in mitigating the actions of adenine-induced CKD in diabetic and non-diabetic rats.

## Figures and Tables

**Table 1:** Effect of metformin (MF) treatment on some physiological parameters in diabetic and non-diabetic rats with adenine (A)-induced chronic kidney disease (CKD).

Parameters/Treatments	Control	A	STZ	A + STZ	MF	A + MF	STZ + MF	A+ MF + STZ
Body weight Change (%)	14.97 ±0.86	0.26 ± 0.91 ^a^	−26.69 ±3.63 ^a^	−27.55 ±3.89 ^a,b^	2.81 ± 0.92	2.78 ±3.02	−17.47 ± 1.78 ^a,e^	−19.43 ± 5.70 ^a,b,e^
Relative kidney weight (%)	0.57 ± 0.02	0.94 ± 0.03 ^a^	0.98 ± 0.03 ^a^	1.40 ± 0.07 ^a,b,c^	0.58 ± 0.03	0.60 ± 0.04 ^b^	0.84 ± 0.02 ^e^	1.07 ± 0.18 ^a,e^
Water intake (mL)	19.33 ± 1.05	51.0 ±1.91 ^a^	109.17 ± 4.27 ^a^	132.0 ± 10.80 ^a,b^	13.33 ± 1.93	14.67 ± 1.09 ^b^	42.17 ± 5.48 ^a,c,e^	72.33 ± 7.20 ^a,c,d,e^
Urine output (mL)	11.83 ± 0.83	40.33 ± 0.76 ^a^	92.33 ± 2.17 ^a^	111.0 ± 9.07 ^a,b^	6.25 ± 0.57	9.08 ± 0.58 ^b^	29.67 ±4.36 ^a,c,e^	35.83 ± 5.79 ^a,c,d,e^

Values in the table are means ± SEM (*n* = 6). Diabetes was induced by a single streptozotocin (STZ) intraperitoneal injection (55 mg/kg) on the first day of the experiment, three weeks later CKD was induced by feeding A (0.25%) for 35 days, and MF (200 mg/kg) was concurrently given orally to rats. On the 35th day of A and MF treatment, the rats were placed in metabolic cages to collect urine. Differences between the groups were assessed by one-way analysis of variance (ANOVA) followed by Bonferroni’s multiple comparison test, where *p* < 0.05. ^a^ denotes significant difference between control group vs. different groups; ^b^ denotes significant difference between the group given A alone vs. other groups treated with A; ^c^ denotes significant difference between the group given STZ alone vs. other groups treated with STZ; ^d^ denotes significant difference between the group given A + STZ vs. other groups treated with A + STZ; ^e^ denotes significant difference between the group given MF only vs. other groups treated with MF.

**Table 2:** Effect of metformin (MF) treatment on some plasma parameters in diabetic and non-diabetic rats with adenine (A)-induced chronic kidney disease (CKD).

Parameters/Treatments	Control	A	STZ	A + STZ	MF	A + MF	STZ + MF	A+ STZ + MF
Urea (mmol/L)	3.0 ± 0.61	18.0 ± 1.57 ^a^	14.1 ± 1.56 ^a^	24.2 ± 3.32 ^a^	1.1 ± 0.18	5.8 ± 1.38 ^b^	5.4 ± 1.26 ^c^	7.8 ± 1.54 ^b–e^
Creatinine(μmol/L)	20.5 ± 1.6	58.2 ± 5.1 ^a^	50.3 ± 8.8 ^a^	61.9 ± 5.4 ^a^	11.7 ± 2.5	37.3 ± 6.3 ^b,d,e^	32.5 ± 5.4 ^d,e^	38.2 ± 4.5 ^b,d,e^
Uric acid (μmol/L)	20.8 ± 1.2	52.4 ± 6.9 ^a^	35.4 ± 1.7 ^a^	52.9 ± 3.3 ^a,c^	26.4 ± 4.2 ^a^	23.3 ± 2.8 ^b^	19.8 ± 2.4 ^c^	23.9 ± 3.0 ^b–,d^
Phosphorus (mmol/L)	0.54 ± 0.08	1.52 ± 0.19 ^a^	0.97 ± 0.28	1.42 ± 0.18 ^a^	0.66 ± 0.07	0.71 ± 0.12 ^b^	0.70 ± 0.10	0.80 ± 0.05 ^b,d^
Calcium (mmol/L)	0.80 ± 0.08	0.37 ± 0.11 ^a^	0.54 ± 0.04 ^a^	0.47 ± 0.10 ^a^	0.81 ± 0.06 ^a^	0.53 ± 0.05 ^a,e^	0.64 ± 0.05	0.57 ± 0.06
IS (μmol)	4.2 ± 0.30	34.8 ± 1.68 ^a^	19.6 ± 1.20 ^a^	39.2 ± 1.81 ^a,c^	4.1 ± 0.24 ^a^	22.4 ± 1.09 ^a,b^	14.4 ± 0.94 ^a,c^	31.9 ± 2.40 ^a,d,e^
Adiponectin (µg/mL)	3.39 ± 0.14	8.91 ± 0.86 ^a^	6.56 ± 0.22 ^a^	9.11 ± 0.80 ^a,c^	2.56 ± 0.42	4.44 ± 0.41 ^b^	4.49 ± 0.26	5.51 ± 0.61 ^b,d,e^
Cystatin C (ng/mL)	9.63 ± 1.66	25.0 ± 2.45 ^a^	14.2 ± 1.43	24.3 ± 2.11 ^a,c^	7.01 ± 0.73	12.0 ± 2.12 ^b,e^	7.39 ± 1.03	11.4 ± 2.14 ^b,d^
NGAL (ng/mL)	27.9 ± 2.17	80.1 ± 7.61 ^a^	44.8 ± 2.76 ^a^	74.3 ± 3.92 ^a,c^	23.3 ± 0.95	40.7 ± 1.62 ^b,e^	29.3 ± 3.55	36.0 ± 3.12 ^b,d^

Values in the tables are means ± SEM (*n* = 6). Diabetes was induced by a single streptozotocin (STZ) intraperitoneal injection (55 mg/kg) on the first day of the experiment, three weeks later CKD was induced by feeding A (0.25%) for 35 days, and MF (200 mg/kg) was concurrently given orally to rats. On the 36th day of treatment, the rats were sacrificed to collect plasma. NGAL: Neutrophil gelatinase-associated lipocalin; IS: Indoxyl sulfate. Differences between the groups were assessed by one-way analysis of variance (ANOVA) followed by Bonferroni’s multiple comparison test, where *p* < 0.05. ^a^ denotes significant difference between control group vs. different groups; ^b^ denotes significant difference between the group given A alone vs. other groups treated with A; ^c^ denotes significant difference between the group given STZ alone vs. other groups treated with STZ; ^d^ denotes significant difference between the group given A + STZ vs. other groups treated with A + STZ; ^e^ denotes significant difference between the group given MF only vs. other groups treated with MF.

**Table 3:** Effect of metformin (MF) treatment on some urine parameters in diabetic and non-diabetic rats with adenine (A)-induced chronic kidney disease (CKD).

Parameters/Treatments	Control	A	STZ	A + STZ	MF	A + MF	STZ + MF	A+ MF + STZ
Creatinine (µmol/L)	4258.7 ± 80.5	908.3 ± 166.7 ^a^	294.3 ± 28.7 ^a^	150.7 ± 38.2 ^a,c^	5326.2 ± 317.6	3578.2 ± 269.5 ^b^	2209.7 ± 660.2 ^a,c,^^d^	2390 ± 408.5 ^a–d^
Creatinine clearance (mL/min)	1.77 ± 0.22	0.44 ± 0.08 ^a^	0.5 ± 0.14 ^a^	0.18 ± 0.03 ^a^	2.67 ± 0.66	0.85 ± 0.30 ^e^	1.40 ± 0.52 ^e^	1.56 ± 0.03 ^a,d,e^
Albumin/creatinine ratio (mg/µmol)	0.52 ± 0.03	4.2 ± 0.7 ^a^	3.23 ± 0.32	5.97 ± 2.48 ^a^	0.69 ± 0.05	0.74 ± 0.10 ^b^	1.61 ± 0.47	1.30 ± 0.58 ^b,d,e^
Osmolality (mOsmol/kg)	2125.0 ± 74	421.0 ± 25 ^a^	752.3 ± 37 ^a^	527.8 ± 58 ^a^	2174.7 ± 91	1247.0 ± 110 ^a,b,e^	1478.0 ± 92 ^a,c,e^	1254.3 ± 32 ^a–e^
NAG activity (nmol/min/mL)	4.42 ± 0.50	22.01 ± 1.99 ^a^	14.42 ± 0.68 ^a^	23.74 ± 2.48 ^a,c^	3.99 ± 0.31	8.86 ± 1.03 ^b,e^	7.74 ± 0.67 ^c^	10.55 ± 1.25 ^a,b,d,e^

Values in the tables are means ± SEM (*n* = 6). Diabetes was induced by a single streptozotocin (STZ) intraperitoneal injection (55 mg/kg) on the first day of the experiment, three weeks later CKD was induced by feeding A (0.25%) for 35 days, and MF (200 mg/kg) was concurrently given orally to rats. On the 35th day of A and MF treatment, the rats were placed in metabolic cages to collect urine. NAG = *N*-acetyl-beta-D-glucosaminidase. Differences between the groups were assessed by one-way analysis of variance (ANOVA) followed by Bonferroni’s multiple comparison test, where *p* < 0.05. ^a^ denotes significant difference between Control group vs. different groups; ^b^ denotes significant difference between A given alone vs. other groups treated with A; ^c^ denotes significant difference between STZ given alone vs. other groups treated with STZ; ^d^ denotes significance of A + STZ alone group vs. other groups treated with A + STZ; ^e^ denotes significance of MF given alone vs. other groups treated with MF.

**Table 4:** Effect of metformin (MF) treatment on some plasma cytokines in diabetic and non-diabetic rats with adenine (A)-induced chronic kidney disease (CKD).

Parameters/Treatments	Control	A	STZ	A + STZ	MF	A + MF	STZ + MF	A + STZ+ MF
IL-1β (pg/mL)	47.4 ± 1.4	158.8 ± 10.5 ^a^	66.8 ± 2.7	174.4 ± 14.4 ^a,c^	30.6 ± 0.9	65.5 ± 4.4 ^b,e^	49.6 ± 3.3	88.7 ± 3.1 ^a,b,d,e^
IL-6 (pg/mL)	49.9 ± 3.9	151.9 ± 5.2 ^a^	110.3 ± 5.8 ^a^	166.6 ± 8.1 ^a^	42.1 ± 8.4	100.6 ± 10.4 ^a,b,e^	63.4 ± 5.2 ^c^	101.9 ± 5.8 ^a,b,d,e^
IL-10 (pg/mL)	630.2 ± 19.3	216.8 ± 10.8 ^a^	360.8 ± 26.9 ^a^	238.2 ± 16.7 ^a^	674 ± 26.0	444.2 ± 30.4 ^a,b,e^	495.8 ± 19.8 ^a,c,e^	399.4 ± 20.2 ^a–e^
TGF-β1 (ng/mL)	32.8 ± 1.0	73.4 ± 3.6 ^a^	51.1 ± 1.6 ^a^	74.8 ± 3.6 ^a,b,c^	30.1 ± 1.6 ^a^	50.3 ± 4.8 ^a,b,e^	33.7 ± 1.6 ^c^	53.8 ± 0.8 ^a,b,d,e^

Values in the tables are means ± SEM (*n* = 6). Diabetes was induced by a single streptozotocin (STZ) intraperitoneal injection (55 mg/kg) on the first day of the experiment, three weeks later CKD was induced by feeding A (0.25%) for 35 days, and MF (200 mg/kg) was concurrently given orally to rats. The plasma was collected 24 h last treatment and used to measure the above. IL-1β = Interleukin-1beta; IL-6 = Interleukin-6; IL-10 = Interleukin-10; TGF-β1 = Transforming growth factor beta-1. Differences between the groups were assessed by one-way analysis of variance (ANOVA) followed by Bonferroni’s multiple comparison test, where *p* < 0.05. ^a^ denotes significant difference between control group vs. different groups; ^b^ denotes significant difference between the group given A alone vs. other groups treated with A; ^c^ denotes significant difference between the group given STZ alone vs. other groups treated with STZ; ^d^ denotes significant difference between the group given A + STZ vs. other groups treated with A + STZ; ^e^ denotes significant difference between the group given MF only vs. other groups treated with MF.

**Table 5:** Effect of metformin (MF) treatment on oxidative status in diabetic and non-diabetic rats with adenine (A)-induced chronic kidney disease (CKD).

Parameters/Treatments	Control	A	STZ	A + STZ	MF	A + MF	STZ + MF	A + MF + STZ
Renal GR (nmol/min/mL)	80.0 ± 2.8	24.7 ± 1.5 ^a^	42.7 ± 5.4 ^a^	38.3 ± 4.9 ^a^	91.6 ± 3.2	57.9 ± 6.4 ^a,b,e^	63.2 ± 3.6 ^e^	53.1 ± 2.9 ^a,b,d,e^
Renal SOD (% relative activity)	100 ± 0.0	34.8 ± 1.8 ^a^	42.1 ± 6.5 ^a^	29.6 ± 2.4 ^a^	115.6 ± 3.1	77.7 ± 5.4 ^a,b,e^	78.6 ± 3.3 ^a,c,e^	52.1 ± 3.8 ^a,b,d,e^
Renal TAC (nmol/µL)	0.85 ± 0.06	0.30 ± 0.01 ^a^	0.43 ± 0.02 ^a^	0.29 ± 0.02 ^a^	1.10 ± 0.04 ^a^	0.70 ± 0.06 ^b,e^	0.79 ± 0.07 ^c,e^	0.69 ± 0.04 ^b–e^
Plasma 8-OHdG (ng/mL)	0.23 ± 0.02	1.06 ± 0.07 ^a^	2.76 ± 0.18 ^a^	4.53 ± 0.33 ^a,b,c^	0.25 ± 0.02 ^a^	0.59 ± 0.03	1.26 ± 0.12 ^a,c,e^	2.26 ± 0.19 ^a,b,e^
Plasma 8-Isoprostane (ng/mL)	6.5 ± 0.06	16.4 ± 0.83 ^a^	19.4 ± 0.85 ^a^	21.2 ± 1.30 ^a,b,c^	6.9 ± 0.07 ^a^	11.7 ± 0.62	13.7 ± 0.81 ^a,c,e^	16.4 ± 0.47 ^a,b,e^

Values in the tables are means ± SEM (*n* = 6). Diabetes was induced by a single streptozotocin (STZ) intraperitoneal injection (55 mg/kg) on the first day of the experiment, three weeks later CKD was induced by feeding A (0.25%) for 35 days, and MF (200 mg/kg) was concurrently given orally to rats. The kidneys were collected 24 h last treatment and used to measure the above. GR = Glutathione reductase, SOD = Superoxide dismutase, TAC = Total antioxidant capacity, 8-OHdG = 8-hydroxy-2-deoxy guanosine. Differences between the groups were assessed by one-way analysis of variance (ANOVA) followed by Bonferroni’s multiple comparison test, where *p* < 0.05. a denotes significant difference between control group vs. different groups; b denotes significant difference between the group given A alone vs. other groups treated with A; c denotes significant difference between the group given STZ alone vs. other groups treated with STZ; ^d^ denotes significant difference between the group given A + STZ vs. other groups treated with A + STZ; ^e^ denotes significant difference between the group given MF only vs. other groups treated with MF.

**Table 6:** Effect of metformin (MF) treatment on histopathological assessment of kidney sections in diabetic and non-diabetic rats with adenine (A)-induced chronic kidney disease (CKD).

Assessment/Treatment	Acute Tubular Necrosis		Fibrosis Index %
	Percentage (%)	Lesion Score	
Control	0.00 ± 0.0	0	5.2
A	81.1 ± 2.22 ^a^	4	34.3
STZ	20.6 ± 2.00 ^a^	2	13.4
A + STZ	78.9 ± 3.30 ^c^	4	33.2
MF	0.6 ± 0.56	0	7.2
A + MF	48.9 ± 1.41 ^a,b,e^	3	26.2
STZ + MF	7.8 ± 1.11 ^a,c,e^	1	6.8
A + STZ + MF	0.00 ± 0.0 ^b,c,d^	0	6.3

Values in the tables are mean ± SEM (*n* = 6). Diabetes was induced by a single intraperitoneal injection of streptozotocin (STZ) (55 mg/kg) on the first day of the experiment, three weeks later CKD was induced by feeding A (0.25%) for 35 days, and MF (200 mg/kg) was concurrently given orally to rats. On the 36th day of treatment, the rats were sacrificed to collect kidney. Differences between the groups were assessed by one-way analysis of variance (ANOVA) followed by Bonferroni’s multiple comparison test, ^a^ denotes significant difference between control group vs. different groups; ^b^ denotes significant difference between the group given A alone vs. other groups treated with A; ^c^ denotes significant difference between the group given STZ alone vs. other groups treated with STZ; ^d^ denotes significant difference between the group given A + STZ vs. other groups treated with A + STZ; ^e^ denotes significant difference between the group given MF only vs. other groups treated with MF.

## Data Availability

The data presented in this study are available on request from Professors Badreldin H. Ali and Abderrahim Nemmar.
